# Characterization of IL-19, -20, and -24 in acute and chronic kidney diseases reveals a pro-fibrotic role of IL-24

**DOI:** 10.1186/s12967-020-02338-4

**Published:** 2020-04-19

**Authors:** Domonkos Pap, Apor Veres-Székely, Beáta Szebeni, Réka Rokonay, Anna Ónody, Rita Lippai, István Márton Takács, András Tislér, Magdolna Kardos, Franz Oswald, Andrea Fekete, Attila J. Szabó, Ádám Vannay

**Affiliations:** 1 MTA-SE, Pediatrics and Nephrology Research Group, Budapest, Hungary; 2 1st Department of Paediatrics, Semmelweis University, Budapest, Hungary; 3 MTA-SE, Lendület Diabetes Research Group, Budapest, Hungary; 4 1st Department of Internal Medicine, Semmelweis University, Budapest, Hungary; 5 2nd Department of Pathology, Semmelweis University, Budapest, Hungary; 6 University Medical Center, Center of Internal Medicine, Department of Internal Medicine I, Ulm, Germany

**Keywords:** Interleukin 20 subfamily, Interleukin 24, Acute kidney disease, Chronic kidney disease, Kidney fibrosis

## Abstract

**Background:**

Recently, the role of IL-19, IL-20 and IL-24 has been reported in renal disorders. However, still little is known about their biological role.

**Methods:**

Localization of IL-20RB was determined in human biopsies and in the kidneys of mice that underwent unilateral ureteral obstruction (UUO). Renal *Il19*, *Il20* and *Il24* expression was determined in ischemia/reperfusion, lipopolysaccharide, streptozotocin, or UUO induced animal models of kidney diseases. The effects of H_2_O_2_, LPS, TGF-β1, PDGF-B and IL-1β on *IL19*, *IL20* and *IL24* expression was determined in peripheral blood mononuclear cells (PBMCs). The extents of extracellular matrix (ECM) and α-SMA, *Tgfb1, Pdgfb,* and *Ctgf* expression were determined in the kidneys of *Il20rb* knockout (KO) and wild type (WT) mice following UUO. The effect of IL-24 was also examined on HK-2 tubular epithelial cells and NRK49F renal fibroblasts.

**Results:**

IL-20RB was present in the renal biopsies of patients with lupus nephritis, IgA and diabetic nephropathy. Amount of IL-20RB increased in the kidneys of mice underwent UUO. The expression of *Il19*, *Il20* and *Il24* increased in the animal models of various kidney diseases. IL-1β, H_2_O_2_ and LPS induced the *IL19*, *IL20* and *IL24* expression of PBMCs. The extent of ECM, α-SMA, fibronectin, *Tgfb1, Pdgfb,* and *Ctgf* expression was lower in the kidney of *Il20rb* KO compared to WT mice following UUO. IL-24 treatment induced the apoptosis and TGF-β1, PDGF-B, CTGF expression of HK-2 cells.

**Conclusions:**

Our data confirmed the significance of IL-19, IL-20 and IL-24 in the pathomechanism of renal diseases. Furthermore, we were the first to demonstrate the pro-fibrotic effect of IL-24.

## Background

The prevalence of chronic kidney disease (CKD) is estimated to be 8–16% worldwide, and is rapidly increasing [1]. The key risk factors of CKD are the chronic civilization diseases including hypertension, diabetes mellitus and autoimmune diseases, but acute kidney injury can also lead to long-term renal damage [2]. Despite the growing medical importance, the pathomechanism of CKD is still not fully understood. However, regardless of its etiology, the pathomechanism of CKD always has an inflammatory component, which leads to the overproduction of pro-fibrotic factors, including transforming growth factor (TGF)–β1, platelet-derived growth factor (PDGF)-B or connective tissue growth factor (CTGF) [3]. These factors are implicated in the activation and proliferation of α smooth muscle actin (α-SMA) positive myofibroblasts (MFs), which are the main effector cells responsible for the excessive production of extracellular matrix (ECM) leading to the destruction of normal kidney architecture, and finally to end-stage renal diseases [4].

The IL-20 subfamily of cytokines is part of the IL-10 family and comprises five related molecules, including IL-19, IL-20, IL-22, IL-24, and IL-26. The IL-20 subfamily of cytokines are mainly produced by immune cells and have been suggested to enhance tissue repair processes [5]. Within the subfamily, IL-19, IL-20, and IL-24 form a distinct group as they share the common IL-20RA/IL-20RB and IL-22RA/IL-20RB receptor heterodimers [6]. However, the distribution of these receptors can vary between tissues, their expression is restricted to the epithelial cells in most organs [5]. Recently, connection between IL-20 subfamily of cytokines and CKD has been proposed. Indeed, increased serum level of IL-19 and IL-24 was demonstrated in diabetic nephropathy and lupus nephritis [7, 8]. Furthermore, our previous genome-wide analysis performed on new-born rat kidneys suggested the possible role of IL-24 in the renal tissue remodeling, as well [9]. Although all these data indicate a role of the IL-20 subfamily in CKD, little is known about the underlying mechanisms. Therefore, in our study, we investigated the role of IL-19, IL-20, and IL-24 in the pathomechanism of CKD. We examined the renal presence of IL-19, IL-20, and IL-24, and their receptors in animal models of different kidney diseases and in renal biopsies of patients with different renal diseases. Experiments were performed to study the regulation of synthesis of IL-19, IL-20 and IL-24. Moreover, the role of investigated cytokines in CKD were examined using *Il20rb* KO mice and HK-2 tubular epithelial cells.

## Methods

### Human kidney biopsies

Human renal biopsy samples were obtained from patients with clinically diagnosed diabetic nephropathy, lupus nephritis, and IgA nephropathy. Histologically intact tumor-free kidney tissues of a patient with renal cancer were used as control (n = 1 in all group). For more detailed description see Additional file 1: Table S2. All human samples were analyzed in a retrospective, anonymized manner, after having received the approval of the Semmelweis University Regional and Institutional Committee of Science and Research Ethics (31224-5/2017/EKU).

### Animals and ethic statement

All animal procedures were approved by the Committee on the Care and Use of Laboratory Animals of the Council on Animal Care at Semmelweis University, Budapest, Hungary (PEI/001/1731-8/2015). In the experiments 6–8 weeks old male C57BL/6J wild type (WT) and *Il20rb* gene knockout (KO) mice (C57BL/6J background) [10], obtained from Franz Oswald, University Medical Center, Ulm, Germany) or 6–8 weeks old male Wistar rats were used. All animals were kept in plastic cages under 12 h dark/light cycle at constant temperature (24 ± 0.2 °C) with free access to standard rodent chow and drinking water. All surgical procedures were performed under total anesthesia by the intraperitoneal (IP) injection of a mixture of 100 mg/kg ketamine and 10 mg/kg xylazine. After the termination of each experiment, kidney and serum samples were collected for the further measurements. The serum creatinine and BUN levels were determined by standard methods using commercially available kits on a Hitachi 912 chemistry analyzer (Roche Hitachi). In UUO experiments, kidney segments were fixed in 4% buffered formaldehyde.

### Unilateral ureteral obstruction induced nephropathy

Unilateral ureteral obstruction (UUO) or sham surgery was performed on WT and *Il20rb* KO mice, as we previously described [9]. Briefly, the left ureter of the mice was isolated by blunt dissection and completely ligated using fine suture material in the UUO group. The sham-operated (control) animals underwent identical surgical procedures without the occlusion of the left ureter (n = 6–7 in each group). Seven (UUO day 7) or 14 days (UUO day 14) after the initiation of UUO, the left kidneys were surgically removed.

### Renal ischemia reperfusion induced acute kidney injury

Renal ischemia/reperfusion (I/R) injury induced acute kidney injury was performed on Wistar rats, as we previously described [11]. Briefly, the left renal pedicle was isolated and occluded with an atraumatic microvascular clamp for 45 min. Before the end of the ischaemic period, the right kidney was removed and the abdomen was closed. The sham-operated (control) animals underwent identical surgical procedure without clamping the left renal artery and vein (n = 5–6 in each group). The rats were sacrificed after 24 h of reperfusion.

### Streptozotocin-induced diabetic nephropathy

Streptozotocin (STZ)-induced diabetes was induced in Wistar rats as previously described [12]. Briefly, the rats received a single IP injection of 65 mg/kg STZ, dissolved in 0.1 M citrate buffer (pH 4.5). Control rats received equivalent volumes of vehicle without STZ (n = 6 in each group). Blood glucose levels were measured three times from the tail vein after an overnight fast. Animals were considered diabetic if their peripheral blood glucose level was above 15 mmol/l 72 h after the STZ injection and remained elevated. The rats were sacrificed 6 weeks after STZ injection.

### Endotoxin-induced acute kidney injury

Lipopolysaccharides (LPS)-induced acute kidney injury was performed on C57BL/6J mice by a single IP injection of 10 mg/kg LPS (*Escherichia coli* serotype 0111:B4; Sigma Aldrich). Control mice were IP injected with the equal volume of vehicle (n = 5–6 in each group). The mice were sacrificed 24 h after LPS injection.

### Recombinant proteins applied for cell culture experiments

Recombinant human interleukin 24 (IL-24), and platelet-derived growth factor B (PDGF-B) were purchased from R&D Systems. Recombinant human interleukin-1 beta (IL-1β) and transforming growth factor beta-1 (TGF-β1) were purchased from Life Technologies. IL-24 was dissolved in sterile phosphate buffered saline (PBS); IL-1β was dissolved in sterile distilled water; TGF-β1 and PDGF-B were dissolved in HCl (4 mmol/l).

### Recombinant proteins

The following recombinant human proteins were used: interleukin-24 (IL-24), platelet-derived growth factor B (PDGF-B) (R&D Systems) interleukin-1 beta (IL-1β) and transforming growth factor beta-1 (TGF-β1) (Life Technologies). IL-24 was dissolved in sterile phosphate buffered saline (PBS); IL-1β in sterile distilled water; TGF-β1 and PDGF-B in HCl (4 mmol/l).

### Cell culture

Human proximal tubular epithelial cells (HK-2) and normal rat kidney fibroblast cells (NRK-49F) were purchased from American Type Culture Collection (ATCC). Both cell lines were cultured in Dulbecco’s modified Eagle’s medium (Gibco, Life Technologies) supplemented with 10% fetal bovine serum (FBS) (Gibco, Life Technologies) and 1% Penicillin–Streptomycin Solution (Sigma-Aldrich) in humidified 95% air and 5% CO_2_ at 37 °C. Before the experiments, the medium was replaced with DMEM without FBS for 24 h. For real-time RT-PCR, flow cytometric and Western blot analysis HK-2 and cells were seeded into 6-well plates at a density of 5 × 10^5^ cells/well (n = 5–6 well/treatment group) and treated with IL-24 (100 ng/ml) for 24 h.

### Peripheral blood mononuclear cells (PBMCs)

PBMCs from adult control and from patients with diabetic nephropathy CKD were isolated by density gradient centrifugation using Histopaque-1077 (Sigma-Aldrich). After isolation, the cells were placed into RPMI 1640 medium (ATCC) supplemented with 10% FBS and 1% Penicillin–Streptomycin Solution in humidified 95% air and 5% CO_2_ at 37 °C. For real time RT-PCRs, PBMCs were seeded into 24 well plates at a density of 5 × 10^5^ cells/well (n = 5–6 well/treatment group) and treated either with IL-1β (100 ng/ml), TGF-β1 (5 ng/ml), PDGF-B (10 ng/ml), LPS (10 ng/ml), or H_2_O_2_ (25 µmol/l) for 24 h. Vehicle-treated cells served as controls.

### MTT cell proliferation and viability assay

For MTT assays, HK-2 and NRK-49F cells were seeded into 96-well plates at a density of 4 × 10^3^ cells/well (n = 5 well/treatment group), and treated with IL-24 (1, 10 or 100 ng/ml) for 24 h. The assay was performed using Cell Proliferation Kit I (Roche Diagnostics) according to the manufacturer’s recommendations. Absorbance was recorded at 570 nm and at 690 nm as background using a Hidex Chameleon Microplate Reader (Triathler, Plate Chameleion, 300SL Lablogic Systems) using the MikroWin 2000 software.

### LDH cytotoxicity assay

For LDH assays, HK-2 and NRK-49F cells were seeded into 96-well plates at a density of 4 × 10^3^ cells/well (n = 5 well/treatment group) and treated with IL-24 (1, 10 or 100 ng/ml) for 24 h. LDH assay was performed as previously described [13]. All reagents were purchased from Sigma-Aldrich. Absorbance was recorded at 570 nm and at 690 nm as background in a Hidex Chameleon Microplate Reader using the MikroWin 2000 software.

### SiriusRed collagen detection assay

For the SiriusRed assay, NRK-49F cells were seeded into 96-well plates at a density of 10^4^ cells/well (n = 5 well/treatment group), and treated with IL-24 (100 ng/ml) and TGF-β1 (5 ng/ml) for 48 h. The assay was performed as previously described [14]. All reagents were purchased from Sigma-Aldrich. Absorbance was determined at 544 nm and at 690 nm as background by Hidex Chameleon Microplate Reader using the MikroWin 2000 software.

### Immunohistochemical analysis

Immunohistochemistry was performed on paraffin-embedded 5 μm thick tissue sections fixed in formalin (4%, pH 7.4). Slides were deparaffinized in xylene, rehydrated in graded ethanol series, and washed in distilled water. After that, sections were incubated with primary IL-20RB specific antibody (ab124332; rabbit, 1:100, Abcam) for 1 h at room temperature (RT) followed by repeated washing in TBS. Secondary antibody [HISTOLS^®^-R anti-rabbit Detection Systems (Histopathology Ltd.)] was applied for 30 min at RT, followed by repeated washing in TBS. The sections were incubated with 3,3ʹ-Diaminobenzidine (HISTOLS^®^-DAB chromogen/Substrate System, Histopathology Ltd.), washed in distilled water, counterstained with haematoxylin, and finally washed in tap water. No first antibody control was carried out with the omission of primary antibody. Sections were then dehydrated, cleared in xylene, and mounted with permanent mounting medium (DPX Mountant for histology, Sigma-Aldrich). Images were taken with a Zeiss AxioImager A1 Light Microscope (Carl Zeiss GmbH) using 40× objective from each kidney cross-sections with Panoramic Viewer (3DHistech Ltd.).

### Immunocytochemistry

HK-2 and NRK-49F cells were seeded in chambers (Sarstedt) and cultured for 24 h in 37 °C. After repeated washing with PBS, the slides were permeabilized with Cytofix/Cytoperm (BD Pharmingen) for 15 min at RT, then washed again, and incubated with primary antibody specific for IL-20RB (ab124332, rabbit, 1:100, Abcam) for 1 h at RT. The slides were then washed with WashPerm solution and incubated with the corresponding Alexa Fluor 568 secondary antibody (A10042, anti-rabbit, 1:200, Invitrogen) for 30 min (min) at RT in darkness and counterstained with Hoechst 33342 (1:2000, Sigma-Aldrich). Finally, slides were coverslipped with Vectashield fluorescent mounting medium (Vector Laboratories). Appropriate controls were performed by omitting the primary antibodies to assure their specificity and to avoid autofluorescence. Slides were analysed with a Nikon C2 confocal laser scanning microscope system.

### Histological analysis

Formalin-fixed paraffin-embedded kidney samples were cut into 4‑μm sections and stained with Masson’s trichome and SiriusRed staining. The sections were scanned, and on average 15 non-overlapping areas were randomly selected under 200× magnification from each kidney section. The analysis of the Masson’s trichrome and SiriusRed stained areas was performed with ImageJ (The National Institutes of Health). The results were expressed as a percentage of the positively stained area of Masson’s trichrome and SiriusRed staining.

### RNA isolation, reverse transcription and real-time RT-PCR

The total RNA was isolated from the kidney samples, HK-2, NRK-49F, or PBMC cells by Geneaid Total RNA Mini Kit (Geneaid Biotech). RNA was reverse-transcribed using Maxima First Strand cDNA Synthesis Kit for RT-qPCR (Life Technologies) to generate first-stranded cDNA. The mRNA expression of the investigated genes was determined by real-time PCR using LightCycler 480 SYBR Green I Master enzyme mix (Roche Diagnostics) on a Light Cycler 480 system (Roche Diagnostics). The nucleotide sequences, annealing temperatures of the primer pairs, and the resulting PCR product lengths are shown in Additional file 1: Table S1. Relative mRNA expressions were determined by comparison with glyceraldehyde-3-phosphate dehydrogenase (*GAPDH*) or ribosomal protein lateral stalk subunit P0 (*RPLP0*) as internal control using the ∆∆Ct method [15]. Data were normalized and presented as the ratio of their control values.

### Protein isolation and Western blot analysis

Kidney samples or HK-2 cells were homogenized in lysis buffer, containing protease inhibitor cocktail (P8340, Sigma-Aldrich). The protein concentration of the homogenised samples was determined by detergent-compatible protein assay (Bio-Rad Laboratories). The samples were denatured and separated on 4–20% gradient SDS polyacrylamide gel (20 μg protein/lane), then transferred to nitrocellulose membranes. The nitrocellulose membranes were blocked with 5% non-fat milk in tris-buffered saline (TBS) for 1 h at RT. The membranes were incubated overnight at 4 °C with antibodies specific for IL-20Rβ (ab124332; rabbit, 1:1000, Abcam), fibronectin (ab2413, rabbit, 1:1000, Abcam), α-SMA (A2547, mouse, 1:10,000, Sigma-Aldrich) or CTGF (sc-14939, goat, 1:1000, Santa Cruz Biotechnology) or GAPDH (sc-32233, mouse, 1:2000, Santa Cruz Biotechnology). After repeated washing with TBS containing 0.05% Tween-20 and 1% non-fat milk, the membranes were incubated with horseradish peroxidase-conjugated secondary antibodies (sc-2005, 1:2000 anti-mouse, sc-2020 anti-goat 1:2000, sc-2004, anti-rabbit, 1:2000 Santa Cruz Biotechnology) or Alexa Fluor 488 secondary antibody (A11001, anti-mouse, 1:200, Invitrogen) for 1 h at RT. Bands of interest were detected using enhanced chemiluminescence detection (Luminol Reagent, GE Healthcare) or fluorescence detection and quantified by densitometry (VersaDoc, Quantity One Analysis software; Bio-Rad Laboratories) as integrated optical density after background subtraction. Relative protein levels were determined by comparison with GAPDH as internal control. Data were normalized and presented as the ratio of their control values.

### Flow cytometry

HK-2 cells were incubated with Fixation/Permeabilization solution (BD Bioscience) for 10 min at RT. After permeabilization, the cells were washed with Perm/Wash™ Buffer (BD Bioscience) and incubated with TGF-β1 (sc-146, rabbit, 1:50, Santa Cruz Biotechnology) and PDGF-B (sc-7878, rabbit, 1:50, Santa Cruz Biotechnology) specific antibody for 30 min at RT. The cells were subsequently washed with Perm/Wash™ Buffer and incubated with Alexa Fluor 568 secondary antibody (A10042, anti-rabbit, 1:200, Invitrogen) for 30 min (min) at RT in darkness. Appropriate controls were performed omitting the primary antibodies to assure their specificity and to avoid autofluorescence. After that, the cells were washed with Perm/Wash™ Buffer and resuspended in PBS. The flow cytometric analysis was carried out using a FACSAria cytometer (BectonDickinson). The mean fluorescence intensity (MFI) values of each sample were normalized and presented as the ratio of their control values.

### Statistical analysis

The statistical evaluation of data was performed using the GraphPad Prism 6.01 software. Mann–Whitney U-test was used to determine differences between two groups. Multiple comparisons of the data were performed using Kruskal–Wallis test with Dunnett correction. p ≤ 0.05 were considered significant. Values were expressed as mean ± SD.

## Results

### Renal expression of *Il19*, *Il20*, *Il24* and their receptors following UUO

**Fig. 1:**
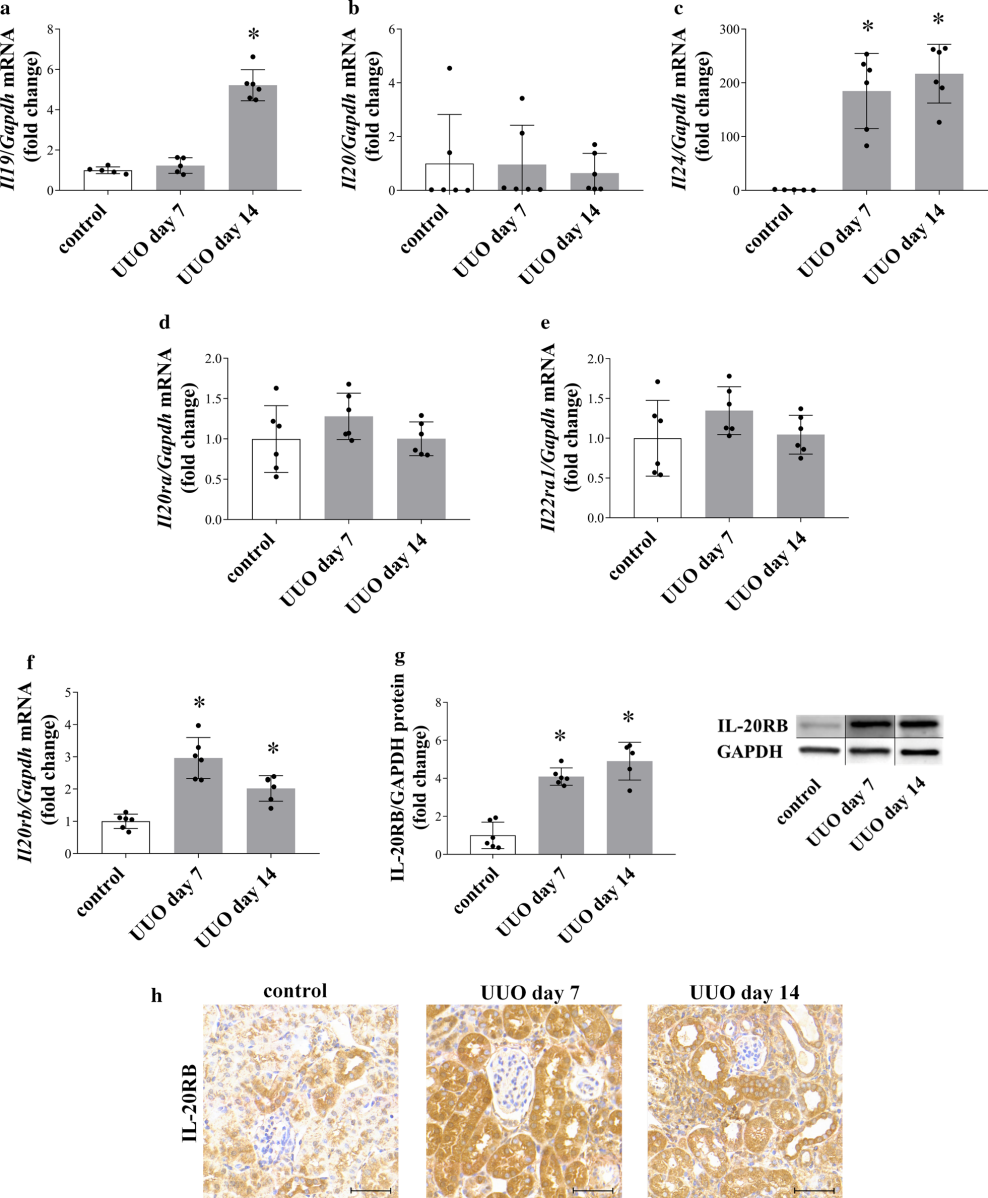
Renal expression of *Il19*, *Il20*, *Il24* and their receptors following unilateral ureteral obstruction (UUO). Renal mRNA expression of *Il19* (**a**), *Il20* (**b**), *Il24* (**c**), *Il20ra* (**d**)*, Il20rb* (**f**) and *Il22ra1* (**e**) of mice underwent UUO and that of controls was determined by real-time RT-PCR in comparison with *Gapdh* as internal control. The renal protein amount of IL-20RB of WT mice underwent UUO and that of controls was measured by Western blot analysis in comparison with GAPDH as internal control (**g** and Additional file 1: Figure S4a). Localization of IL-20RB (brown; **h**) was investigated by immunohistochemical staining in the kidney of mice underwent UUO and in that of controls. Values were expressed as mean ± SD. *n* = 5–6 in each group; *p < 0.05 vs. control (Kruskal–Wallis test). Scale bar: 50 µm (**h**)

To investigate the expression of *Il19*, *Il20*, *Il24* and their receptors in the kidneys of mice underwent UUO real time PCRs were performed. The mRNA expression of *Il19* was increased on day 14, and *Il24* was increased on day 7 and day 14 days after the onset of UUO, respectively (Fig. 1a–c). Similarly, the mRNA expression and protein level of IL-20RB increased on the 7th and 14th day following UUO (Fig. 1f, g). The mRNA expression of *Il20*, *Il20ra* and *Il22ra1* remained unchanged during the whole experiment (Fig. 1b, d, e). Moreover, immunhistological staining was performed to determine the renal localization of IL-20RB receptor subunit. We observed strong IL-20RB immunopositivity in the tubular epithelial and interstitial cells of both sham operated control and UUO kidneys (Fig. 1h).

### The expression of *Il19, Il20* and *Il24* in the kidney of I/R-, LPS- or STZ-induced animal models of kidney diseases

**Fig. 2:**
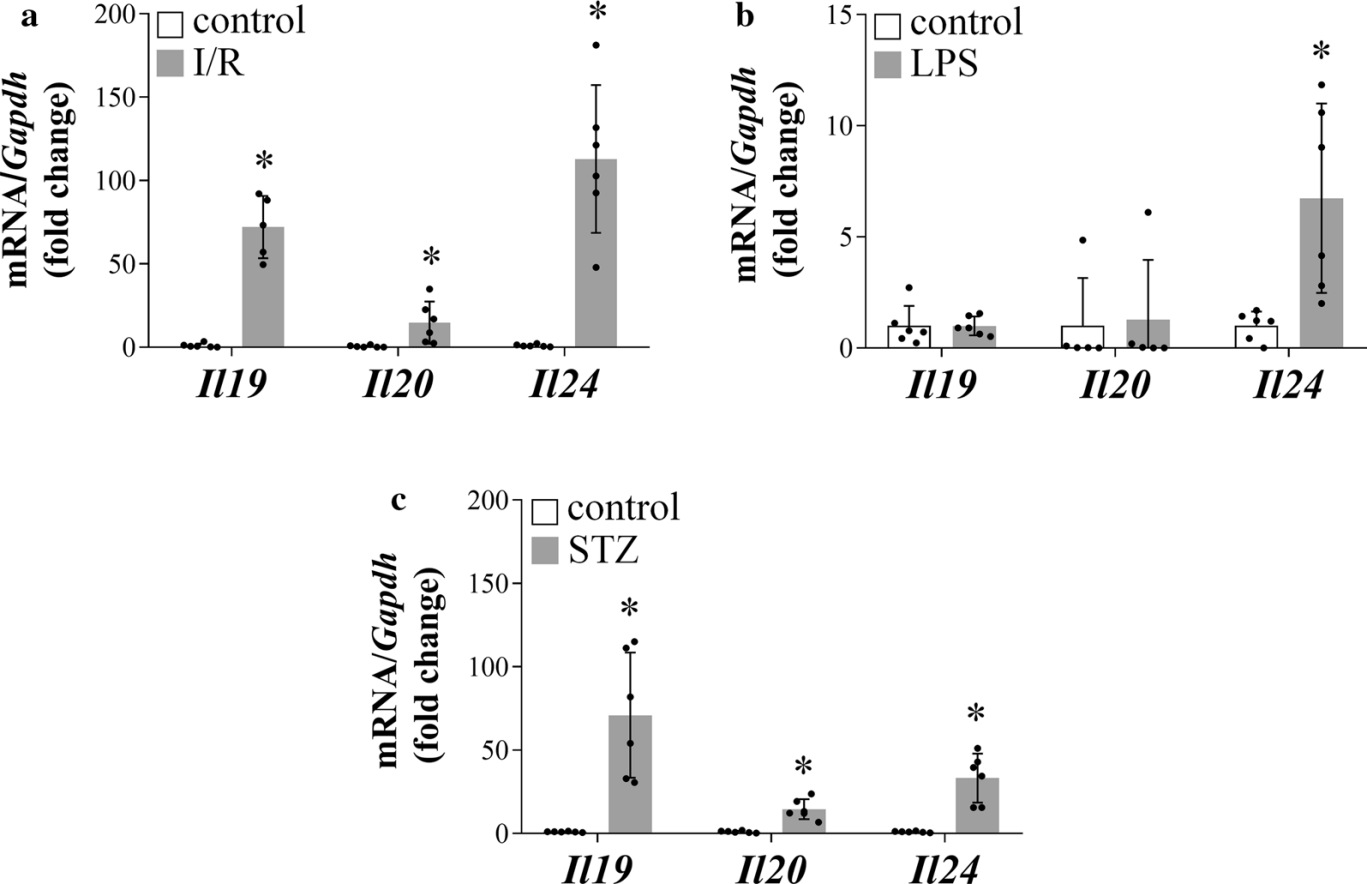
The expression of *Il19, Il20* and *Il24* in the kidney of ischemia/reperfusion (I/R), lipopolysaccharide (LPS) or streptozotocin (STZ) induced animal models of kidney diseases. The renal mRNA expression of *Il19*, *Il20*, and *Il24* of rats with I/R (**a**) or LPS (**b**) induced acute kidney disease, or that of STZ-induced diabetic rats (**c**) was determined by real-time RT-PCR in comparison with *Gapdh* as internal control. Values were expressed as mean ± SD. *n* = 5–6 in each group; *p < 0.05 vs. control (Mann–Whitney U-test). Scale bar: 50 µm

To study the role of IL-19, IL-20 and IL-24 in renal pathophysiology we investigated their expression in various animal models leading to impaired kidney function as demonstrated by the increased serum creatinine and BUN levels (Additional file 1: Figure S2). The mRNA expression of *Il19*, *Il20* and *Il24* increased in the kidney of rats with I/R-induced acute kidney injury and in the kidney of diabetic rats as well (Fig. 2a, c). While the mRNA expression of *Il24* increased, the mRNA expression of *Il19* and *Il20* remained unchanged in the kidneys of LPS-treated mice (Fig. 2b).

### Presence of IL-20RB in human kidney biopsies

**Fig. 3:**
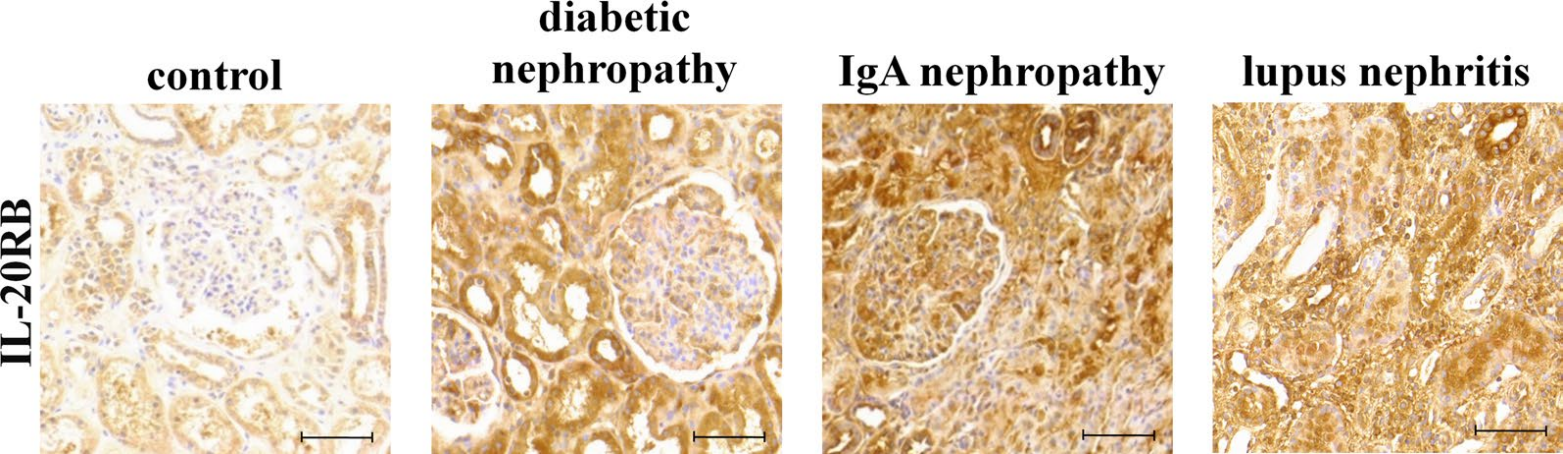
The presence of IL-20RB in human kidney biopsies. The localization of IL-20RB was determined by immunohistochemical staining (brown) in the kidney biopsy samples obtained from control and patients with diabetic and IgA nephropathy or lupus nephritis. Scale bar: 50 µm

In order to demonstrate the presence of IL-20RB in the human kidney samples immunohistological stainings were performed. We found evident IL-20RB immunopositivity in tubular epithelial and glomerular cells of human kidney biopsies obtained from controls and patients with diabetic and IgA nephropathy or lupus nephritis (Fig. 3).

### Expression of *IL19, IL20* and *IL24* of PBMCs

**Fig. 4:**
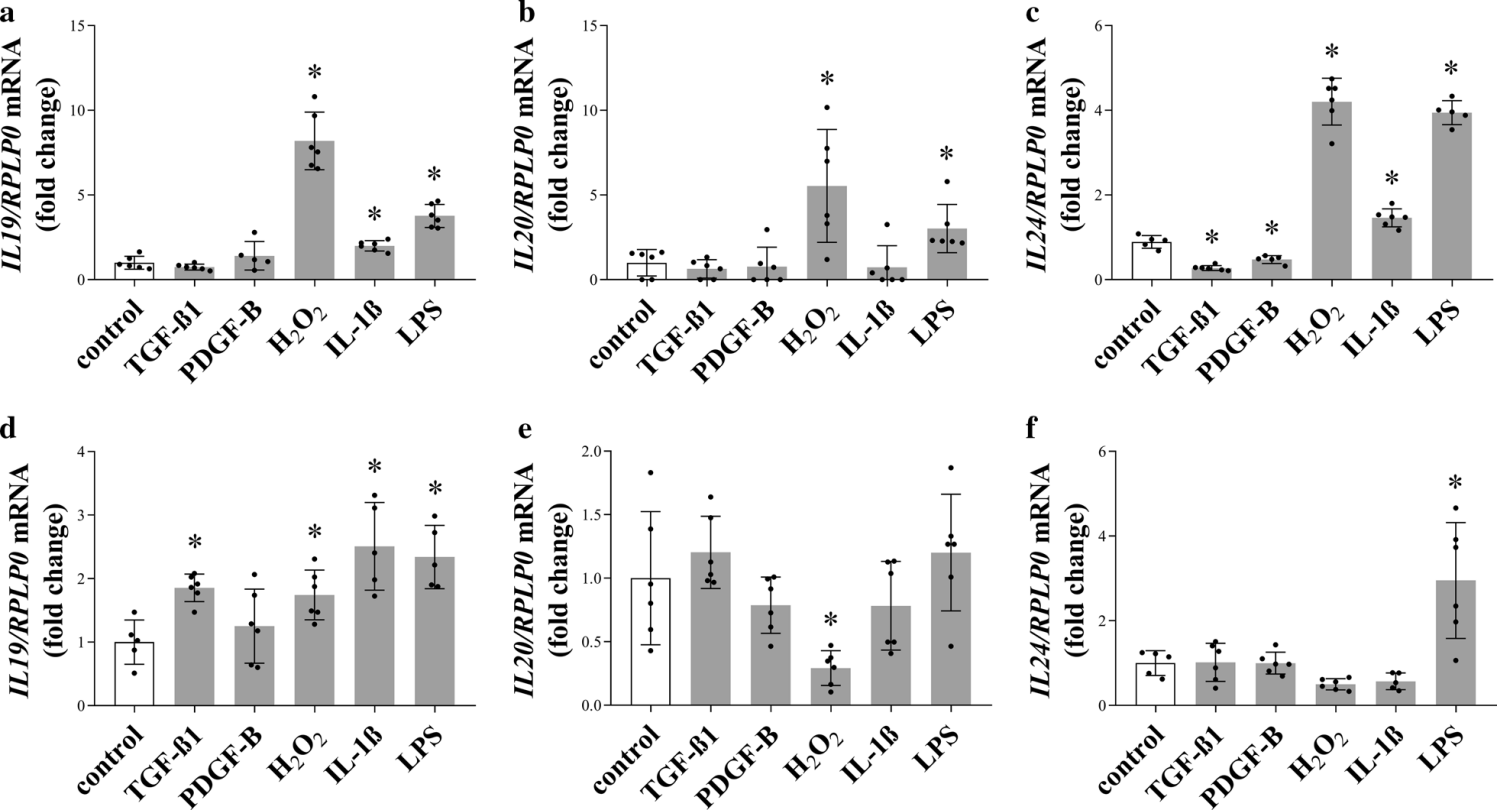
The effect of fibrosis related factors on the expression of *IL19*, *IL20,* and *IL24* in PBMCs. The mRNA expression of *IL19*, *IL20,* and *IL24* in healthy (**a**–**c**) and CKD-derived (**d**–**f**) PBMCs after TGF-β1, PDGF-B, H_2_O_2_, IL-1β, and LPS treatment was measured by real-time RT-PCR in a comparison with *RPLP0* as internal control. Values were expressed as mean ± SD. *n* = 5–6 in each group; *p < 0.05 vs. control (Mann–Whitney U-test)

To understand the mechanisms leading to increased production of *IL19*, *IL20* and *IL24* in the injured kidney we investigated the effect of several fibrosis related factors on their expression in healthy and CKD-derived PBMCs, as well. While H_2_O_2_ and LPS treatment increased the mRNA expression of *IL19*, *IL20* and *IL24,* IL-1β treatment increased only that of *IL24,* TGF-β1 and PDGF-B treatment decreased the mRNA expression of *IL24* of PBMCs derived from healthy adults (Fig. 4a–c). In contrast, LPS treatment increased the mRNA expression of *IL19* and *IL24,* TGF-β1, H_2_O_2_ and IL-1β treatment increased that of *IL19*, and TGF-β1 treatment decreased the mRNA expression of *IL20* in CKD-derived PBMCs (Fig. 4d–f).

### Differences in fibrosis markers in the kidney of WT and *Il20rb* KO mice after UUO

**Fig. 5:**
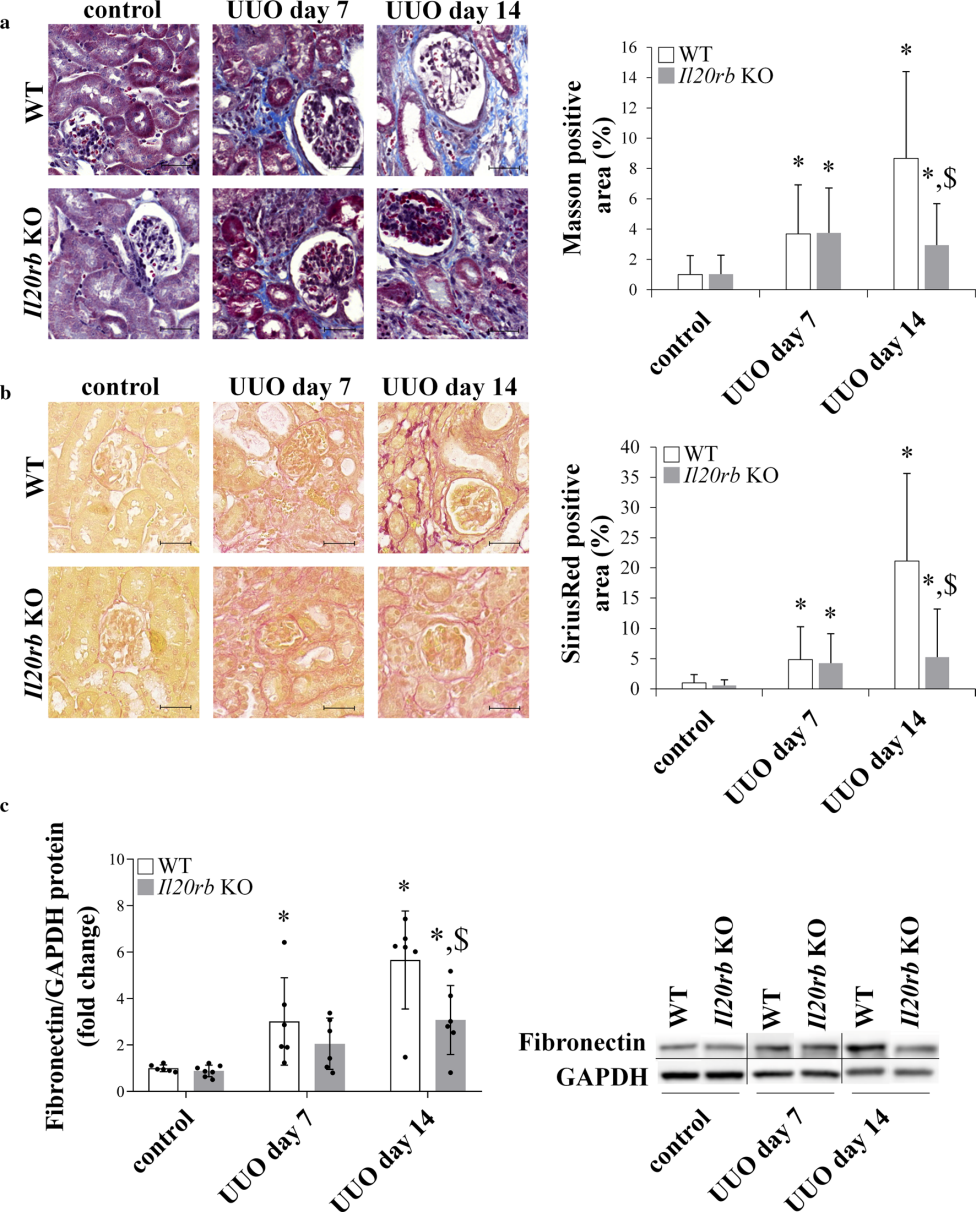

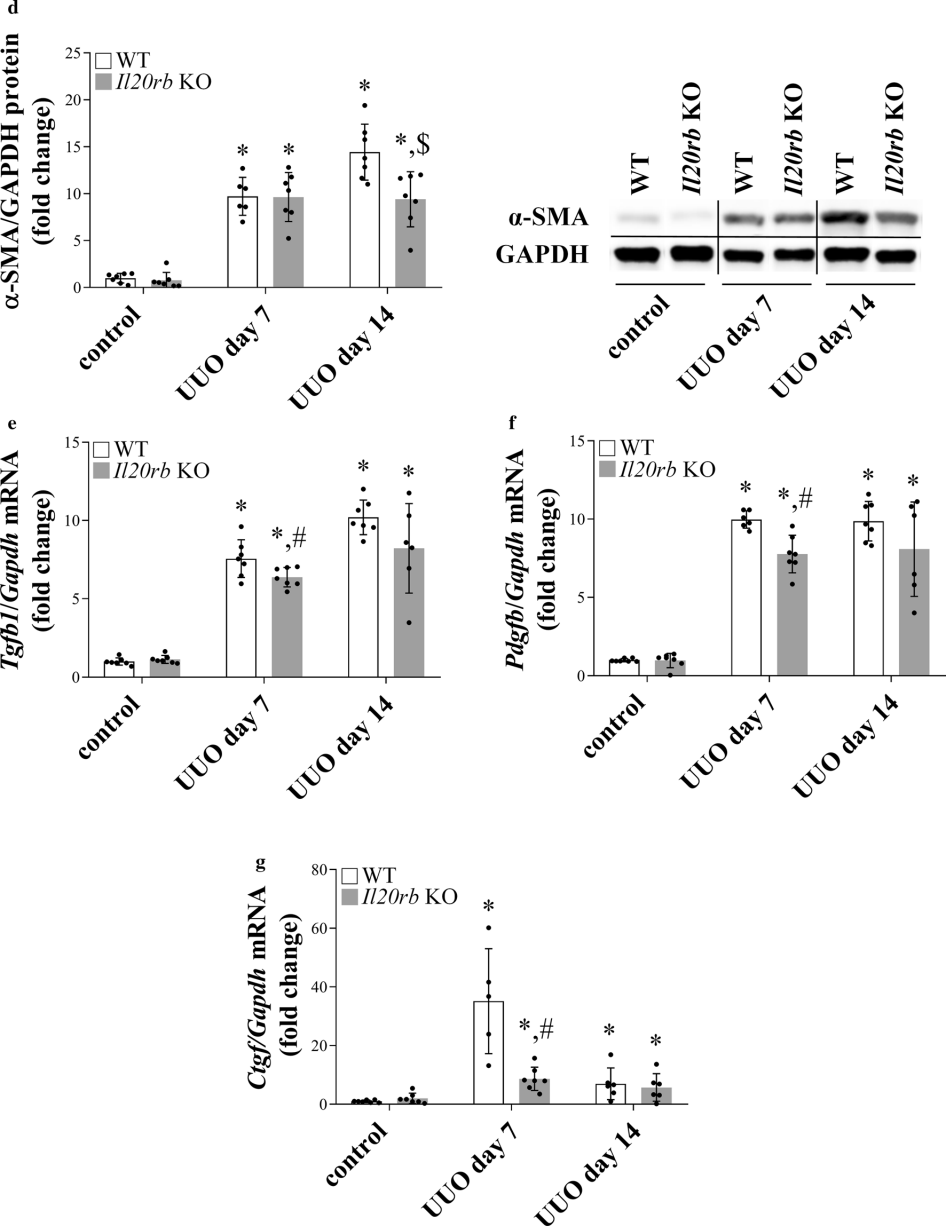
Alteration of fibrosis markers in the kidneys of WT and *Il20rb* KO mice following UUO. The blue areas of the Masson’s trichrome and the red areas of the SiriusRed-stained kidney sections represent the collagen deposits of WT and *Il20rb* KO mice following the onset of UUO (Fig. 3a, b). The protein amount of fibronectin and α-SMA in the kidney tissue of WT and *Il20rb* KO mice after UUO was measured by Western blot analysis in comparison with GAPDH as internal control (**c**, **d** and Additional file 1: Figure S4b, c). The renal mRNA expression of *Tgfb1* (**e**) *Pdgfb* (**f**) and *Ctgf* (**g**) in the kidney tissues of WT and *Il20rb* KO mice was determined by real-time PCR in comparison with *Gapdh* as internal control. Values were expressed as mean ± SD. *n* = 6–7 in each group; *p < 0.05 vs. control (Kruskal–Wallis test); ^#^p < 0.05 vs. WT UUO day 7 (Mann–Whitney U-test); ^$^p < 0.05 vs. WT UUO day 14 (Mann–Whitney U-test). Scale bar: 50 µm (**a**, **b**)

In order to examine the *in vivo* effect of IL-19, IL-20 and IL-24 on renal scarring UUO was performed on *Il20rb* KO and also on WT mice and the renal amount of the markers of tissue remodeling were determined. Renal amount of α-SMA and fibronectin and also the extent of renal Masson’s trichrome and SiriusRed positivity increased in both mouse strains, however it remained unchanged after 7th day in the *Il20rb* KO mice, therefore, finally, the amount of each fibrosis markers was lower in the kidney of the *Il20rb* KO compared to WT mice on day 14 (Fig. 5a–d). The renal mRNA expression of pro-fibrotic growth factors including *Tgfb1*, *Pdgfb* and *Ctgf* also increased in both strain after UUO. However, the expression of these growth factors was lower in the kidney of *Il20rb* KO mice on day 7 following UUO compared to that of WT mice (Fig. 5e–g).

### The effects of IL-24 treatment on the viability and pro-fibrotic growth factor production of HK-2 cells

**Fig. 6:**
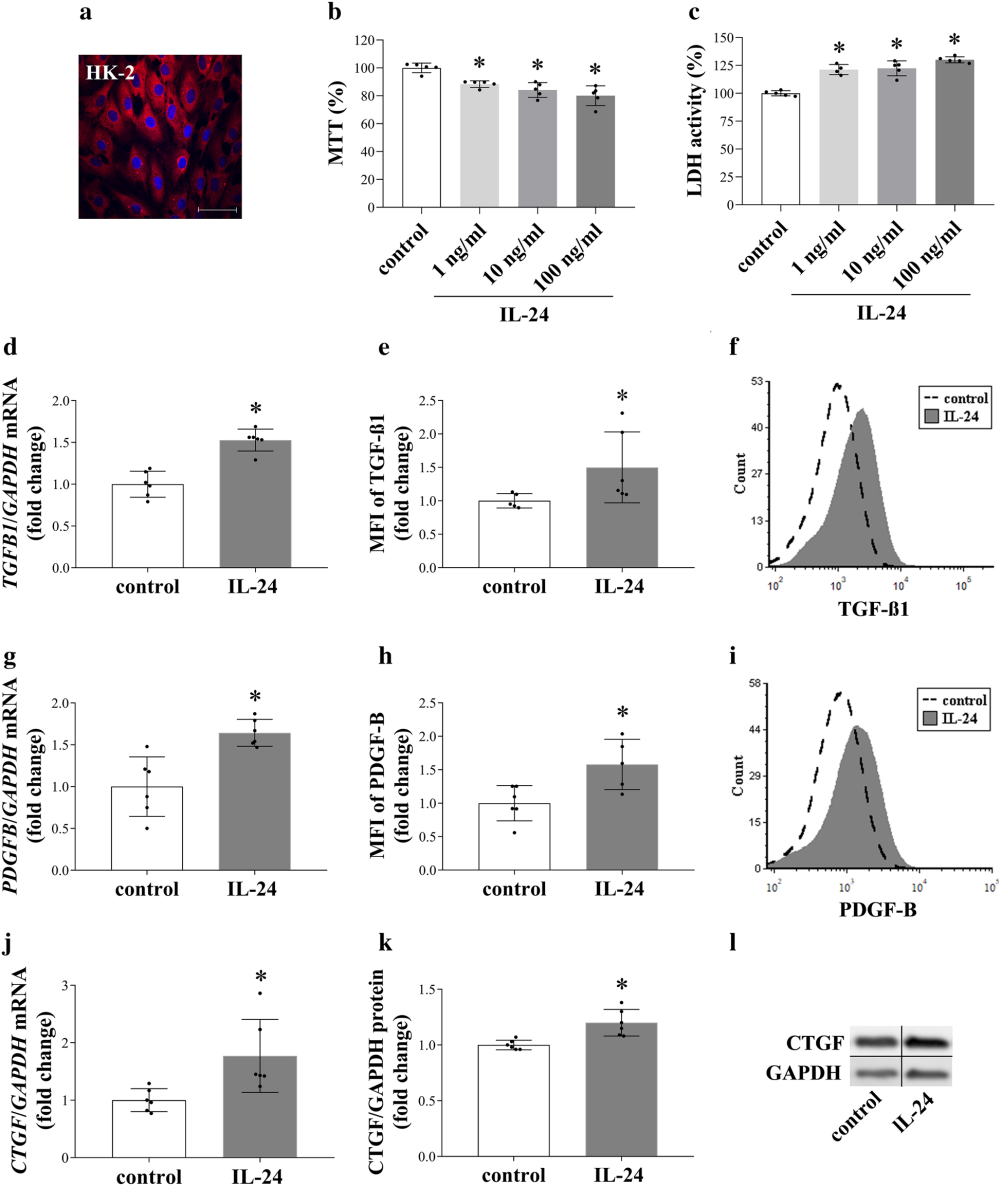
Effect of IL-24 treatment on HK-2 cells. Presence of IL-20RB (red) was determined by immunofluorescence staining on HK-2 cells (**a**). Cell nuclei were counterstained with Hoechst 33342 (blue). Cell viability was determined by MTT (**b**) and LDH (**c**) assays. The mRNA expression of *TGFB1* (**d**) *PDGFB* (**g**) *CTGF* (**j**) after IL-24 treatment (100 ng/ml) was measured by real-time RT-PCR in comparison with *GAPDH* as internal control. The protein level of TGF-β1 (**e**, **f**) PDGF-B (**h**, **i**) after IL-24 treatment (100 ng/ml) was measured by flow cytometry (**g**, **h**). The protein level of CTGF (**k**, **l**) after IL-24 treatment (100 ng/ml) was measured by Western blot analysis in comparison with GAPDH as internal control (**l** and Additional file 1: Figure S4d.) Values were expressed as mean ± SD. *n* = 5–6 in each group; *p < 0.05 vs. control (Mann–Whitney U-test). Scale bar: 50 µm (**a**)

Our in vivo results demonstrated strong IL-20RB positivity on kidney tubular epithelial cells. In line with this data our immunofluorescence staining (Fig. 6a) showed that HK-2 cells also express IL-20RB. Furthermore, our results showed that IL-24 treatment dose dependently decreased viability of HK-2 cells (Fig. 6b). Accordingly, the LDH activity increased in the supernatant of IL-24 treated cells (Fig. 6c). Furthermore, IL-24 treatment increased the mRNA expression of *TGFB1*, *PDGFB* and *CTGF* and protein level of TGF-β1, PDGFB and CTGF of HK-2 cells (Fig. 5d–l). Interestingly, IL-24 treatment did not modulate the viability and ECM production of NRK-49F renal fibroblasts (Additional file 1: Figure S1b, d).

## Discussion

Previously, our research group identified *Il19* and *Il24,* the members of the IL-20 subfamily, as one of the most abundantly expressed genes in the kidneys of new-born rats that underwent UUO [9]. Within the subfamily, IL-19, IL-20, and IL-24 form a distinct group, as they exert their biological activity by binding to IL-20RA/IL-20RB or IL-22RA/IL-20RB receptor heterodimers [6].

So far, no differences in the biological activity of IL-19, IL-20 and IL-24 have been revealed and also the reason of their redundant presence in the kidney is still unexplored.

In the present study we demonstrated that IL-20RB is abundantly present on the epithelial and glomerular cells of patients with CKD of different aetiology, including diabetic and IgA nephropathy, or lupus nephritis (Fig. 3). Furthermore, we found increased presence of IL-20RB on tubular epithelial and glomerular cells in the kidneys of mice underwent UUO (Fig. 1g, h). IL-20RB is a critical component of the IL-20RA/IL-20RB or IL-22RA/IL-20RB receptor heterodimers, it is responsible not only for ligand binding, but also for the formation of the heterodimers, and thus for the biological activity of the receptors [16]. Previous studies found elevated serum level of IL-19 in patients with diabetic nephropathy [7]. Moreover, positive correlation was demonstrated between serum IL-24 level and the severity of lupus nephritis and renal ischemic/reperfusion injury [8, 17]. Considering the above data strongly implies the role of the IL-20 subfamily in the pathomechanism of different renal diseases. Therefore, we further investigated the presence, regulation, and possible role of the subfamily in the pathomechanism of renal diseases.

In line with our previous study on new-born rats, we found markedly increased expression of *Il19* and *Il24* in the kidneys of adult mice after the onset of UUO (Fig. 1a–c). We also demonstrated the increased renal expression of *Il19, Il20,* and *Il24* in an I/R-induced rat model of acute renal failure (Fig. 2a) and in the STZ-induced rat model of diabetic nephropathy, as well (Fig. 2c). However, only the renal expression of *Il24* increased in the mouse model of LPS-induced acute kidney injury (Fig. 2b). These in vivo experiments further strengthened the hypothesis about the unified importance of the investigated cytokines in the different renal pathologies leading to CKD.

Based on the literary data, PBMCs, including lymphocytes, monocytes and macrophages, are the potential source of IL-20 subfamily cytokines in the kidney; however, the factors regulating synthesis of IL-19, IL-20, and IL-24 in CKD have not been previously clarified [18–20]. Therefore, in the present study, we investigated the role of those factors, including TGF-β1, PDGF-B, IL-1β, H_2_O_2_ and LPS, which play a central role in the pathomechanism of the different animal models of the above mentioned renal diseases (Fig. 4a–f) [21, 22]. We found that the effect of oxidative stress and LPS treatment was the most pronounced on the expression of *IL19*, *IL20*, and *IL24* of PBMCs, originated from healthy adults. Moreover, in accordance with Kunz et al., who demonstrated the IL-1β mediated expression of *IL19* and *IL24* in keratinocytes, we found that IL-1β treatment induced the expression of *IL19* and *IL24* in PBMCs, as well [23]. On the other hand, we found that core factors of tissue remodelling, including TGF-β1 and PDGF-B decreased the *IL24* expression of healthy PBMCs. Interestingly, the regulation of the investigated cytokines showed different pattern in the PBMCs of patients with diabetic nephropathy. While the expression of *IL19* was increased by several factor including TGF-β1, IL-1β, LPS and H_2_O_2_, the expression of *IL20* or *IL24* remained mainly unchanged, only LPS treatment induced the expression of *IL24*. The response of PBMCs of different origin to TGF-β1 treatment is of particular interest. While TGF-β1 or PDGF-B treatment decreased the expression of *IL24* of healthy PBMCs, TGF-β1 increased the synthesis of *IL19* in the PBMCs of CKD patients. Previously, in accordance with our present results we demonstrated that TGFβ treatment decrease the IL-24 expression of PBMCs derived from children with coeliac disease [24]. Speculating about the possible molecular mechanism previously, it has been shown that TGF-β1 may induce the expression of IL-1 receptor-associated kinase M (IRAK-M), which is a well-known inhibitor of cytokine expression [25]. Moreover, Gorelik et al. found that TGF-β inhibit the expression of cytokines through the inhibition of GATA binding protein 3 (GATA3) expression, which is known to be involved in the regulation of IL-24 expression of the Th2 lymphocytes [26–28]. Taken together our results demonstrate that PBMCs are capable to produce the cytokines of the IL-20 subfamily in response to the prominent mediators of renal diseases, however there is a clear difference between the PBMCs of healthy adults and those of patients with CKD, which may be due to the previous activation of the PBMCs originating from patients with CKD [29]. In the next set of experiments, the effects of the investigated cytokines were further studied in mice lacking IL-20RB. We found that the protein level of α-SMA, which is a marker of the myofibroblasts (MFs), is lower in the kidneys of *Il20rb* KO mice underwent UUO compared to that of WT ones (Fig. 5d). MFs are responsible for the production of collagens and fibronectin, which are the most common components of the scar tissue [30]. Accordingly, we found less increased ECM depositions, and fibronectin level in kidneys of *Il20rb* KO than in those of WT mice underwent UUO (Fig. 5a–c), which implies the pro-fibrotic role of the investigated cytokines. Our results are in line with the previous observations implying the role IL-19 in intestinal and the role of IL-24 in liver fibrosis and wound healing [19, 31, 32]. Moreover, our results are in accordance with Van belle et al. who found that the genetic deficiency of *Il20rb* resulted in less severe contact dermatitis, which is also characterized by increased collagen depositions [33].

Investigating the underlying molecular mechanisms, we found a decreased expression of *Tgfb1,* *Pdgfb* and *Ctgf,* the core factors of renal fibrosis in the kidneys of *Il20rb* KO compared to WT mice after UUO (Fig. 5e–g). All these growth factors play a well-known role in the regulation of tissue remodelling [34]. TGF-β1 and CTGF promote MF differentiation and induce the excessive production of ECM components, including collagens and fibronectin. Similarly, PDGF-B is a mitogenic growth factor inducing the proliferation of MFs [35]. Therefore, it is easy to accept that decreased deposition of ECM is closely related to the decreased amount of the investigated growth factors in *Il20rb* KO compared to WT mice.

Since IL-20RB is markedly present on the renal tubular epithelial cells of different human biopsies and also on that of mice that underwent UUO, further in vitro experiments were performed on HK-2 tubular epithelial cells expressing IL-20RB (Fig. 6a). In these experiments, we focused on the effects of IL-24, the renal expression of which increased to the greatest extent in our in vivo experiments. Indeed, we found that IL-24 treatment induced the death of HK-2 cells in a dose-dependent manner (Fig. 6b, c). Previously, the apoptosis-inducing ability of IL-24 was thought to be specific to cancer cells only, however, in line with our present experiment, Hsu and Li et al., demonstrated that members of the subfamily including IL-19 and IL-20 induce apoptosis of HK-2 kidney epithelial and M1 cortical duct cells [36–38].

Moreover, in line with our in vivo experiments demonstrating that the lack of IL-20RB is associated with decreased synthesis of *Tgfb1*, *Pdgfb* and *Ctgf*, we found that IL-24 treatment induces TGF-β1, PDGF-B and CTGF synthesis of HK-2 tubular epithelial cells in vitro (Fig. 6d–l). Interestingly, IL-24 treatment had no effect either on the proliferation, or on the ECM production of NRK-49F renal fibroblasts (Additional file 1: Figure S1b, d). Therefore, we imply that IL-24 may act indirectly on renal myofibroblasts via an increased epithelial production of the pro-fibrotic growth factors. This hypothesis is also supported by the observation that the extent of the scar tissue was smaller in the kidney of *Il20rb* KO mice underwent UUO compared to that of WT mice. However, since IL-19 and IL-20 signal through the same receptor complexes it is also possible that they exert similar effects on the scar tissue formation, therefore further experiments are needed to clarify their role in the process of renal fibrosis.

## Conclusions

**Fig. 7:**
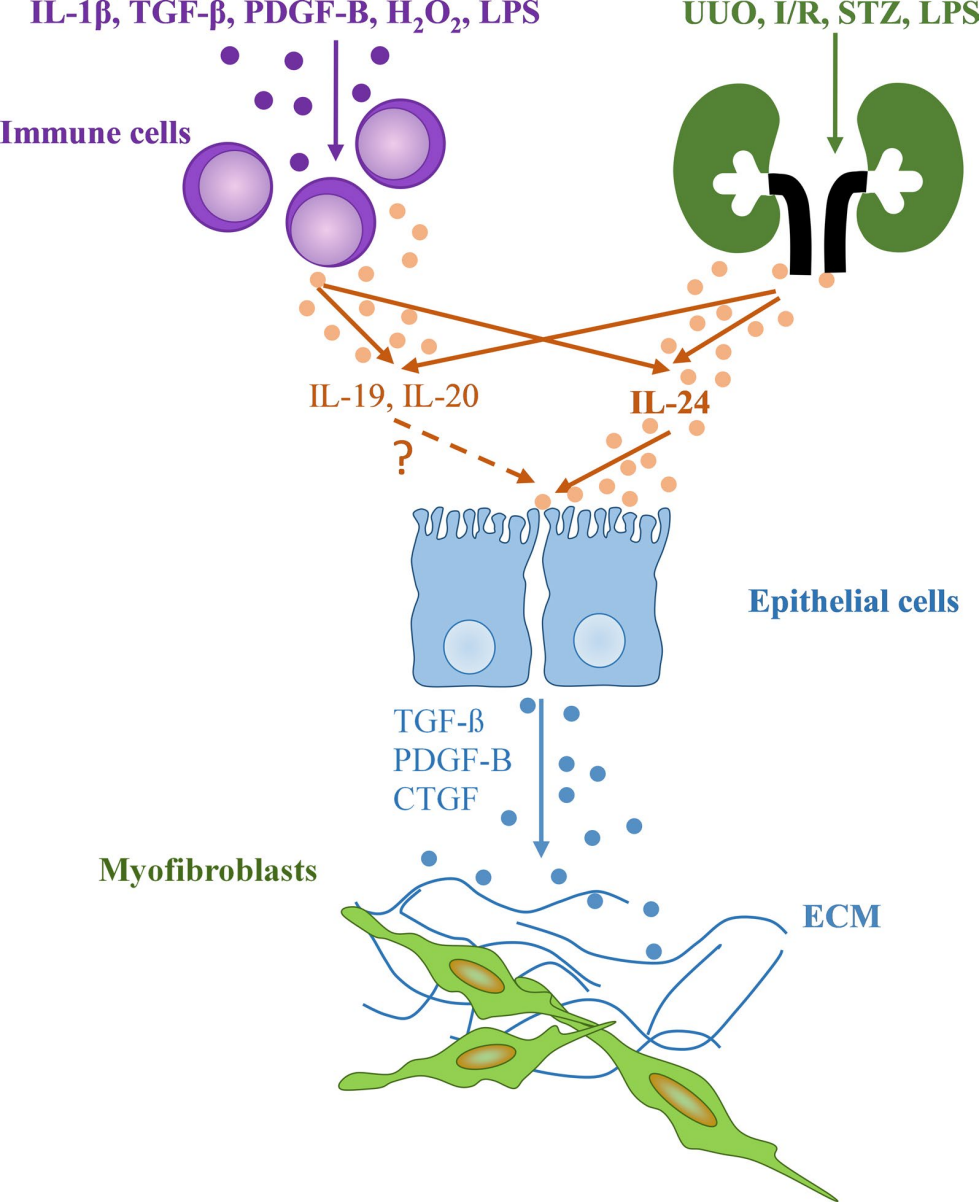
Schematic representation of how the IL-20 cytokine subfamily members may mediate the progression of tissue fibrosis. Our results demonstrated the increased expression of *Il19*, *Il20*, and *Il24* in different renal pathologies. We have shown the role of IL-1β, TGF-β, PDGF-B, H_2_O_2_, LPS on the regulation of *Il19*, *Il20*, and *Il24* expression of the PBMCs. We also demonstrated the presence of their common receptor subunit, IL-20RB on kidney epithelial cells. Moreover, we described the role of IL-20RB in the renal fibrosis. Furthermore, we revealed the effect of IL-24 on profibrotic growth factor production of renal tubular epithelial cells that may lead to activation of renal myofibroblasts

In summary, we showed the presence of IL-20RB in the kidneys of experimental animals, and also in kidney biopsy specimens of patients with diabetic and IgA nephropathy, or lupus nephritis. We also demonstrated the increased production of *Il19*, *Il20*, and *Il24* in the kidney samples from the different animal models of acute and chronic kidney disease. Furthermore, we defined a role of IL-20RB on the renal fibrosis in vivo. Moreover, our experiments described the role of IL-1β, TGF-β1, PDGF-B, LPS, and oxidative stress on the synthesis of IL-19, IL-20 and IL-24 of PBMCs. Finally, we have also shown the effect of IL-24 on the viability and TGF-β1, PDGF-B, and CTGF production of the renal tubular epithelial cells in vitro and in vivo. Our observations raise the possibility that the increased renal production of IL-24 may activate the IL-20RB-associated signaling pathways leading to increased epithelial cell death and an improved synthesis of pro-fibrotic factors (Fig. 7). We hope that our work may contribute to the better understanding of the pathomechanism of CKD, and to the development of new diagnostic and therapeutic approaches to treat CKD.

## Supplementary information

**Additional file 1. Table S1.** Nucleotide sequences of primer pairs, product length, and specific annealing temperatures applied for the real-time reverse transcriptase polymerase chain reaction (RT- PCR) detection; **Table S2.** Description and histological diagnosis of renal biopsy samples obtained from control and CKD patients; **Figure S1.** The effect of IL-24 treatment on NRK-49F cells; **Figure S2.** Effect of ischemia/reperfusion (I/R) (a), lipopolysaccharide (LPS) (b) or streptozotocin (STZ) (c) induced renal injury on kidney function of mice and the hydronephrotic kidney after unilateral ureteral obstruction (UUO) (d); **Figure S3.** Renal expression of Il1b, Il6, Tnfa, Bax, Hmox1, Nqo1, Kim1 and Ngal following LPS induced acute kidney disease; **Figure S4.** Images of entire Western Blot membranes belong to Figure 1/g (a), Figure 4/c (b), Figure 4/d (c) and Figure 5/l (d)

## Data Availability

The datasets used and/or analyzed during the current study are available from the corresponding author on reasonable request.

## Abbreviations

CKD: Chronic kidney diseases
CTGF: Connective tissue growth factor
ECM: Extracellular matrix
FBS: Fetal bovine serum
GAPDH: Glyceraldehyde-3-phosphate dehydrogenase
HCl: Hydrogen chloride
HK-2: Human proximal tubular epithelial cells
I/R: Ischemic reperfusion
IL-: Interleukin
IP: Intraperitoneal
KO: Knockout
LDH: Lactate dehydrogenase
LPS: Lipopolysaccharide
MF: Myofibroblast
MFI: Mean fluorescence intensity
NRK49F: Normal rat kidney fibroblast cell
PBMC: Peripheral blood mononuclear cell
PBS: Phosphate buffered saline
PDGF-B: Platelet-derived growth factor B
RPLP0: Ribosomal protein lateral stalk subunit P0
RT: room temperature
SD: Standard deviation
SDS: Sodium dodecyl sulfate
STZ: Streptozotocin
TBS: Tris buffered saline
TGF-β1: Transforming growth factor beta-1
UUO: Unilateral ureteral obstruction
WT: Wild type
