# Supplementary material for: Characterization of IL-19, -20, and -24 in acute and chronic kidney diseases reveals a pro-fibrotic role of IL-24
Source: J Transl Med. 2020 Apr 19;18:172. doi: 10.1186/s12967-020-02338-4 (PMC7168946; doi:10.1186/s12967-020-02338-4)
Supplement: Supplementary file 1 — Additional file 1. Table S1. Nucleotide sequences of primer pairs, product length, and specific annealing temperatures applied for the real-time reverse transcriptase polymerase chain reaction (RT- PCR) detection; Table S2. Description and histological diagnosis of renal biopsy samples obtained from control and CKD patients; Figure S1. The effect of IL-24 treatment on NRK-49F cells; Figure S2. Effect of ischemia/reperfusion (I/R) (a), lipopolysaccharide (LPS) (b) or streptozotocin (STZ) (c) induced renal injury on kidney function of mice and the hydronephrotic kidney after unilateral ureteral obstruction (UUO) (d); Figure S3. Renal expression of Il1b, Il6, Tnfa, Bax, Hmox1, Nqo1, Kim1 and Ngal following LPS induced acute kidney disease; Figure S4. Images of entire Western Blot membranes belong to Figure 1/g (a), Figure 4/c (b), Figure 4/d (c) and Figure 5/l (d) [file 12967_2020_2338_MOESM1_ESM.docx]

**Table S1.**

Nucleotide sequences of primer pairs, product length, and specific annealing temperatures applied for the real-time reverse transcriptase polymerase chain reaction (RT- PCR) detection.

| **Gene** | **Primer pairs** | | **Product lenght** | **T_a_** |
| --- | --- | --- | --- | --- |
|  |  |  |  |  |
| **human *IL19*** | F: | 5'-CAC CTT CCC AAA TGT CAC TAT-3' | 95 bp | 51 °C |
|  | R: | 5'-ACG CCA GGA GGT TCT TG-3' |  |  |
| **human *IL20*** | F: | 5'-CGC CAA TTC CTT TCT TAC CAT CAA-3' | 157 bp | 56 °C |
|  | R: | 5'-TTC CCC CAA AGC CTT CAC AAC T-3' |  |  |
| **human *IL24*** | F: | 5'-AGG CGG TTT CTG CTA TTC C-3' | 55 bp | 48 °C |
|  | R: | 5'-GAG CTG CTT CTA CGT CCA ACT-3' |  |  |
| **human *RPLP0*** | F: | 5'-GGG GGA ATG TGG GCT TTG TGT T-3' | 206 bp | 59 °C |
|  | R: | 5'-GGT GCC CCT GGA GAT TTT AGT GGT-3' |  |  |
| **human GAPDH** | F: | 5'-AGC AAT GCC TCC TGC ACC ACC AA-3' | 159 bp | 60 °C |
|  | R: | 5'-GCG GCC ATC ACG CCA CAG TTT-3' |  |  |
| **human TGFB1** | F: | 5'-GCG TGC GGC AGC TGT ACA TTG ACT-3' | 173 bp | 60 °C |
|  | R: | 5'-CGA GGC GCC CGG GTT ATG C-3' |  |  |
| **human PDGFB** | F: | 5'-AGA TGG GGC CGA GTT GGA CCT GAA-3' | 163 bp | 62 °C |
|  | R: | 5'-GCG CCG GGA GAT CTC GAA CAC CT-3' |  |  |
| **human CTGF** | F: | 5'-CTC CAC CCG GGT TAC CAA TGA CAA-3' | 228 bp | 58 °C |
|  | R: | 5'-CAG CAT CGG CCG TCG CTA CAT ACT-3' |  |  |
| **mouse Il19** | F: | 5'-CTG GGC ATG ACG TTG ATT CTT T-3' | 79 bp | 51 °C |
|  | R: | 5'-TGA GGC GCA TGT CCA CAG A-3' |  |  |
| **mouse Il20** | F: | 5'-AGC CTC GCC AAC TCC TTT CTT A-3' | 84 bp | 52 °C |
|  | R: | 5'-TGF TTC TTC CCC ACA ATG ACA T-3' |  |  |
| **mouse *Il24*** | F: | 5'-TGT GGG AGG CCT TCT GGA CTG T-3' | 133 bp | 57 °C |
|  | R: | 5'-AGC AGG CTG TGG GCA AGG TAA C-3' |  |  |
| **mouse *Il20ra*** | F: | 5'-ACG CGC AGT TCC TTG TGT CTT CT-3' | 185 bp | 56 °C |
|  | R: | 5'-TCC CGC ATT TAG AGG CAT TCA G-3' |  |  |
| **mouse *Il20rb*** | F: | 5'-GGG CCC CAG TTT GAG TTC CTT GTG-3' | 119 bp | 59 °C |
|  | R: | 5'-GCC CCC GGT TCC ATG GTT TCT A-3' |  |  |
| **mouse *Il22ra1*** | F: | 5'-GTG GGC CCG CTA GCA CCT CTG ACA-3' | 177 bp | 60 °C |
|  | R: | 5'-CCG CGC TGA CCG CCG TGA C-3' |  |  |
| **mouse *Tgfb1*** | F: | 5'-GTG CGG CAG CTG TAC ATT GAC TTT-3' | 239 bp | 60 °C |
|  | R: | 5'-GGC TTG CGA CCC ACG TAG TAG AC-3' |  |  |
| **mouse *Pdgfb*** | F: | 5'-CTG GGC GCT CTT CCT TCC TCT C-3' | 170 bp | 60 °C |
|  | R: | 5'-CCA GCT CAG CCC CAT CTT CAT C-3' |  |  |
| **mouse *Ctgf*** | F: | 5'-CCT CCG TCG CAG GTC CCA TCA GC-3' | 250 bp | 65 °C |
|  | R: | 5'-GGG GAG CCG AAA TCG CAG AAG AGG-3' |  |  |
| **mouse *Il1b*** | F: | 5'-GCC ACC TTT TGA CAG TGA TGA GAA-3' | 136 bp | 55 °C |
|  | R: | 5'-GAT GTG CTG CTG CGA GAT TTG A-3' |  |  |
| **mouse *Il6*** | F: | 5'-AAC CAC GGC CTT CCC TAC TTC A-3' | 155 bp | 55 °C |
|  | R: | 5'-TGC CAT TGC ACA ACT CTT TTC TCA-3' |  |  |
| **mouse *Tnfa*** | F: | 5'-GGG CCA CCA CGC TCT TCT GTC TA-3' | 83 bp | 56 °C |
|  | R: | 5'-GAG AGG GAG GCC ATT TGG GAA CTT-3' |  |  |
| **mouse *Bax*** | F: | 5'-GGA TGA TTG CCG CCG TGG ACA CAG-3' | 215 bp | 60 °C |
|  | R: | 5'-CAA CAG CCG CTC CCG GAG GAA GTC-3' |  |  |
| **mouse *Hmox1*** | F: | 5'-AGA CCG CCT TCC TGC TCA ACA TT-3' | 160 bp | 57 °C |
|  | R: | 5'-GAT TTT CCT CGG GGC GTC TCT G-3' |  |  |
| **mouse *Nqo1*** | F: | 5'-TGG CCG AAC ACA AGA AGC-3' | 262 bp | 56 °C |
|  | R: | 5'-TGA ATC GGC CAG AGA ATG AC-3' |  |  |
| **mouse *Kim1*** | F: | 5'-AAGGGGCATATTTCAGAAGGAGA-3' | 165 bp | 53 °C |
|  | R: | 5'-TCTTGGAGGACGTGTGGGAAT-3' |  |  |
| **mouse *Ngal*** | F: | 5'-GCCAGGCCCAGGACTCAACTCA-3' | 104 bp | 60 °C |
|  | R: | 5'-GTACCACCTGCCCCGGAACTGAT-3' |  |  |
| **mouse *Gapdh*** | F: | 5'-ATC TGA CGT GCC GCC TGG AGA AAC-3' | 164 bp | 60 °C |
|  | R: | 5'-CCC GGC ATC GAA GGT GGA AGA GT-3' |  |  |
| **rat *Il19*** | F: | 5’-AGT TGG CGA TTC TGC TGA TTC TCC -3’ | 239 bp | 57°C |
|  | R: | 5’-TTC TGT GGA CAT GCG CCT CCT G -3’ |  |  |
| **rat *Il20*** | F: | 5'-CCT AGT TCC CCC AAA GCC TTC A-3' | 166 bp | 57°C |
|  | R: | 5'-GCC TCG CAA ATT CTT TTC TAA TAC-3' |  |  |
| **rat *Il24*** | F: | 5'-AAG TGT CCG GCT GTT GAA-3' | 219 bp | 52°C |
|  | R: | 5'-AGC ATG GCA TTG TTC TTA CT-3' |  |  |
| **rat *Gapdh*** | F: | 5’-GGT GAA GGT CGG AGT CAA CG -3’ | 159 bp | 60°C |
|  | R: | 5’-CAA AGT TGT CAT GGA TGA CC -3’ |  |  |

**Table S2.**

| **Sample** | **Gender** | **Age (year)** | **Biopsy type** | **Diagnosis** | **Histological description and additional data** |
| --- | --- | --- | --- | --- | --- |
| Control patient | male | 67 | surgical | Renal carcinoma | Histologically intact tumor-free kidney region. |
| Patient 1. | female | 37 | needle | IgA-nephropathy | Strong mesangial IgA positivity, tubular atrophy and focal segmental glomerulosclerosis. |
| Patient 2. | female | 26 | needle | Systemic lupus erythematosus | Mesangial proliferative glomerulonephritis and "full house" pattern glomerular immunoglobulin deposits. Stage: Class III (A/C) |
| Patient 3. | male | 60 | needle | Diabetic nephropathy | Glomerulosclerosis and mesangial thickening with moderate interstitial fibrosis |

**Table S2. Description and histological diagnosis of renal biopsy samples obtained from control and CKD patients.**

**Additional Results**

The effect of IL-24 on the viability and collagen production of NRK-49F cells

We also examined the ability of IL-24 to regulate viability and ECM synthesis of renal fibroblasts. IL-20RB immunopositivity was demonstrated on NRK-49F kidney cells (Figure 6/a). IL-24 treatment did not modulate the viability of NRK-49F cells (Figure 6/b). Accordingly, IL-24 treatment did not affect the LDH release of NRK-49F cells (Figure 6/c) either. IL-24 treatment had no effect on the collagen synthesis of NRK-49F cells (Figure 6/d).


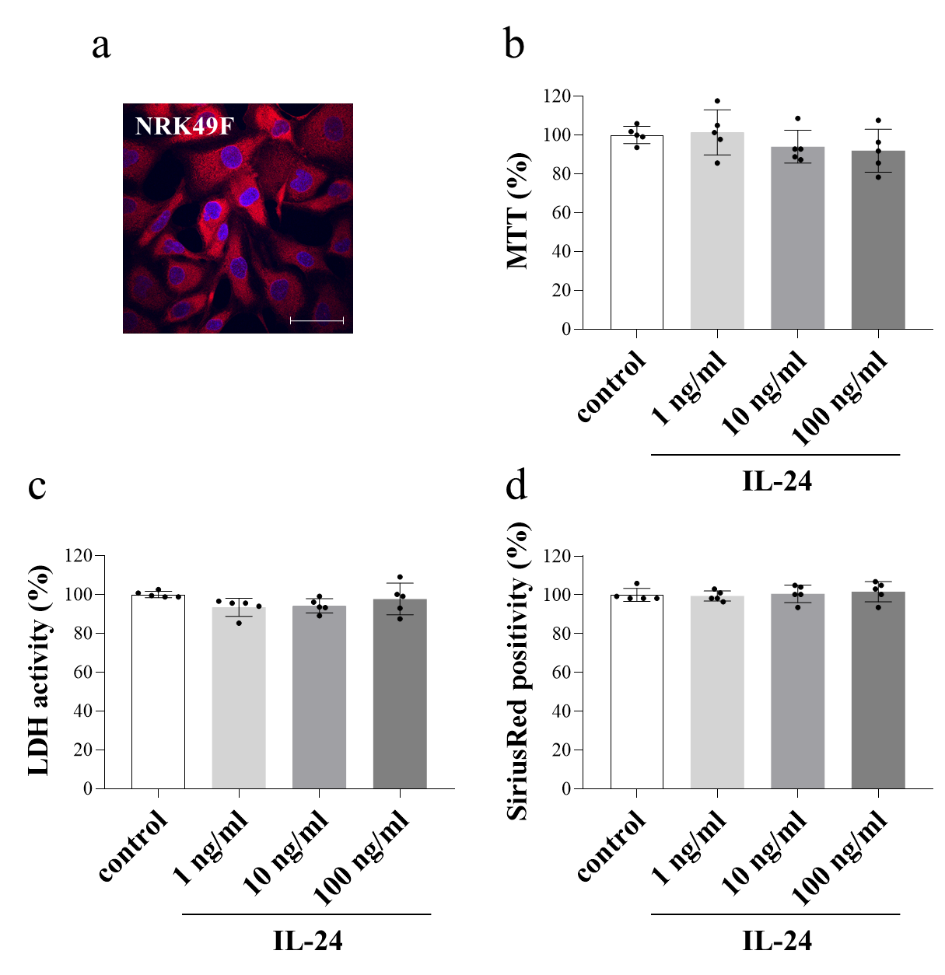


**Figure S1. The effect of IL-24 treatment on NRK-49F cells.** The presence of IL-20RB (red) on NRK-49F cells was determined by immunofluorescence staining (a). Cell nuclei were counterstained with Hoechst 33342 (blue). Cell viability was investigated by MTT (b) and LDH (c) assays. Collagen deposition was investigated after IL-24 treatment by SiriusRed assay (d). Values were expressed as mean±SD. *n*=5 in each group; Scale bar: 50 µm (a).

**Figure S2.**


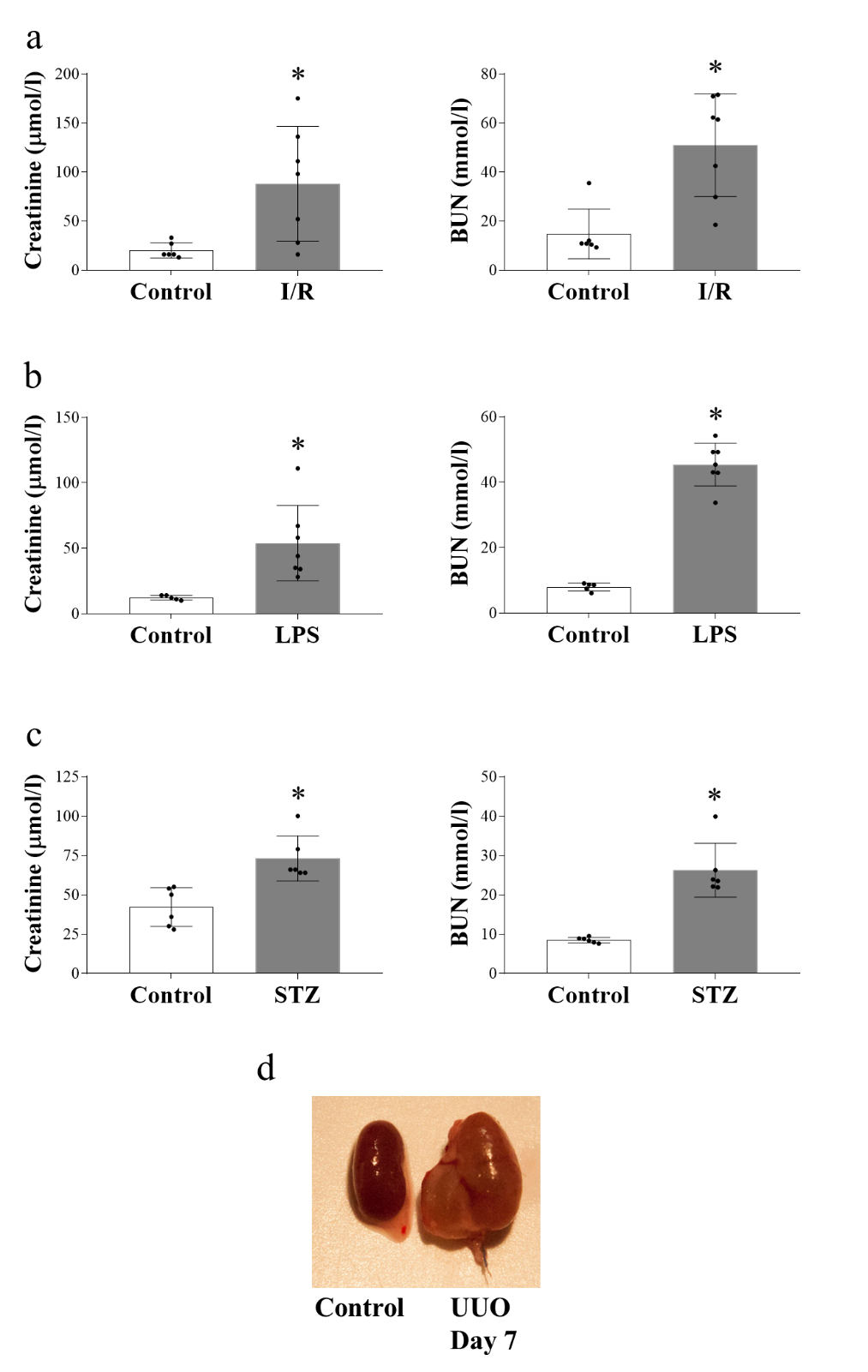


**Figure S2. Effect of ischemia/reperfusion (I/R) (a), lipopolysaccharide (LPS) (b) or streptozotocin (STZ) (c) induced renal injury on kidney function of mice and the hydronephrotic kidney after unilateral ureteral obstruction (UUO) (d).** The serum creatinine and BUN levels were determined by standard methods using commercially available kits on a Hitachi 912 chemistry analyzer (Roche Hitachi). The demonstrative image (d) was taken about a control and obstructed kidney 7 days after the onset of UUO. Values were expressed as mean±SD. n=5-6 in each group; *p<0.05 vs. control (Mann-Whitney U-test)

**Figure S3.**


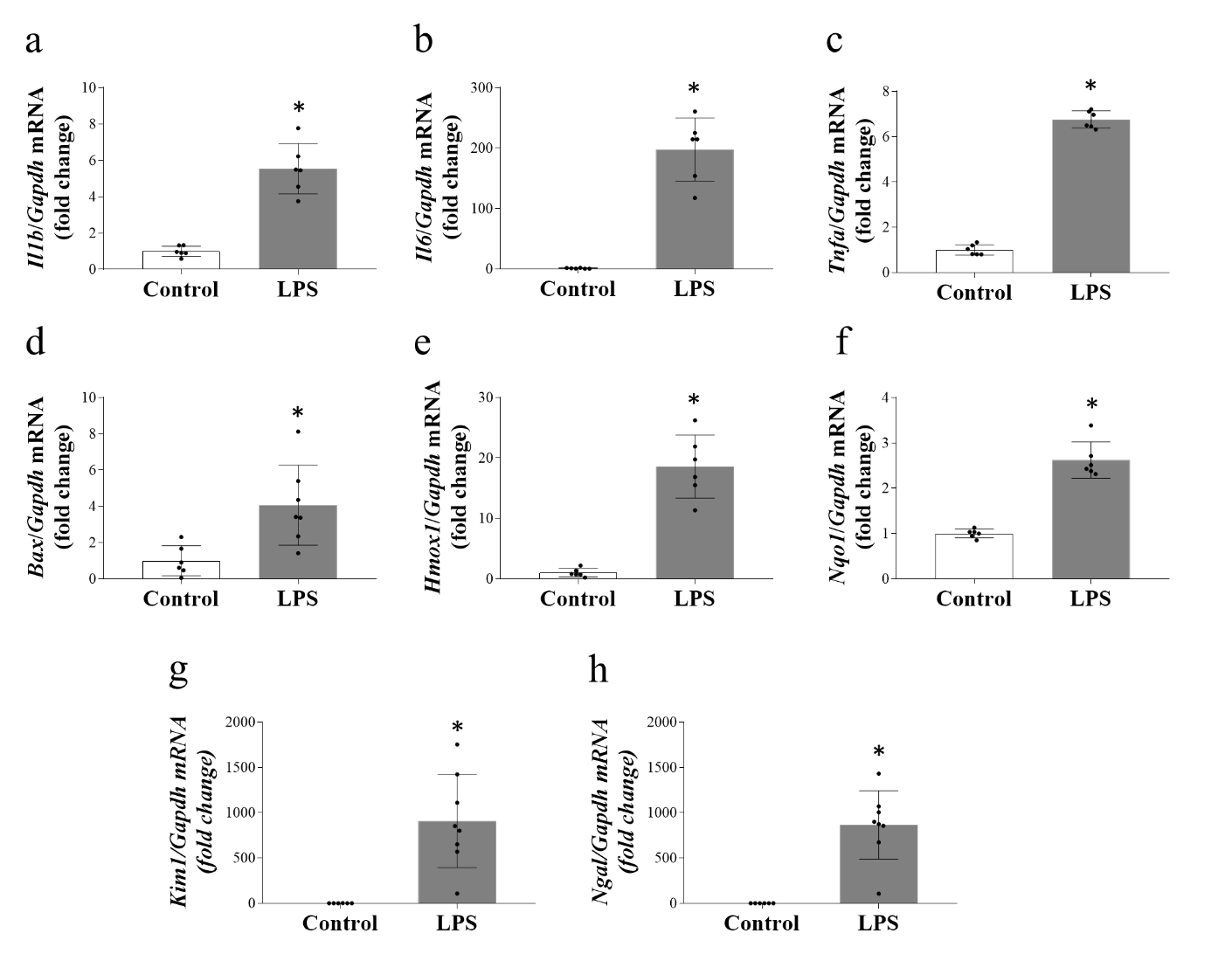


**Figure S3. Renal expression of *Il1b*, *Il6*, *Tnfa*, *Bax*, *Hmox1*, *Nqo1*, *Kim1* and *Ngal* following LPS induced acute kidney diseases.** Renal mRNA expression of *Il1b* (a)*, Il6* (b)*, Tnfa* (c)*,* *Bax* (d)*, Hmox1* (e)*, Nqo1* (f)*, Kim1* (g) *and Ngal* (h) of mice intraperitonealy injected with LPS and that of controls was determined by real-time RT-PCR in comparison with *Gapdh* as internal control. Values were expressed as mean±SD. *n*=5-6 in each group; *p<0.05 vs. control (Mann-Whitney U-test).

**Figure S4.**


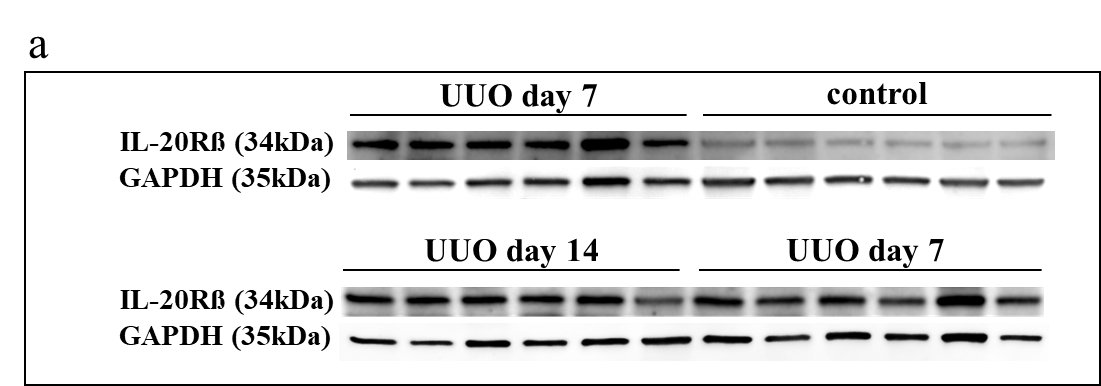


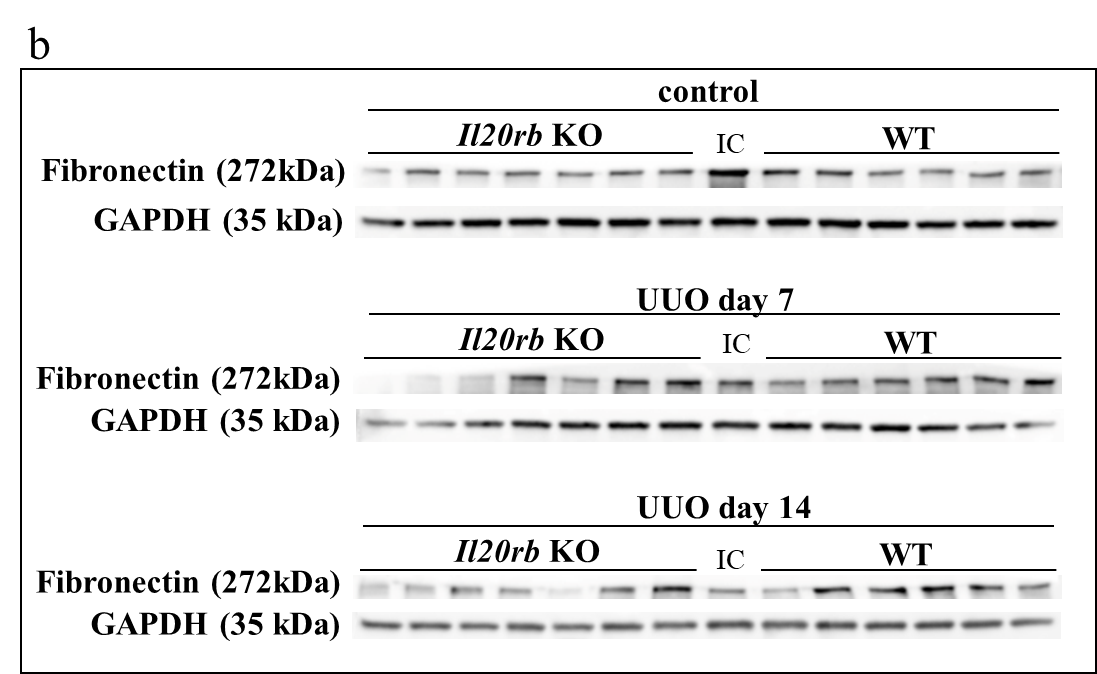


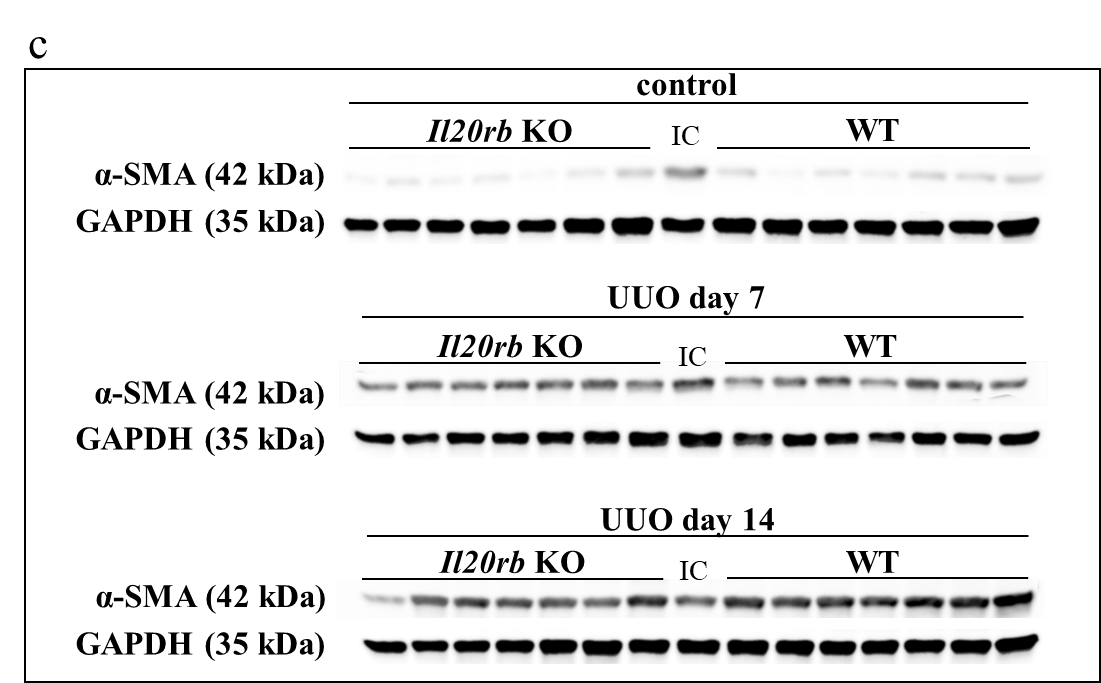


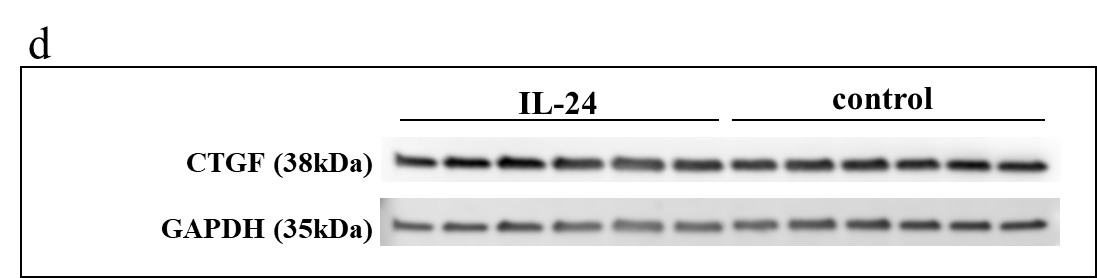


**Figure S4.** Images of entire Western Blot membranes belong to Figure 1/g (a), Figure 4/c (b), Figure 4/d (c) and Figure 5/l (d). Internal control (IC).
